# N-terminally endogenous mKate2-tagged HIM-4 in *Caenorhabditis elegans*

**DOI:** 10.17912/micropub.biology.000386

**Published:** 2021-04-29

**Authors:** Shinji Ihara

**Affiliations:** 1 Faculty of Life and Environmental, Prefectural University of Hiroshima, Hiroshima, Japan

## Abstract

The gene *him-4 *encodes the* C. elegans *homolog of hemicentin, an evolutionarily conserved extracellular matrix protein. Despite being an extracellular matrix protein, mutations in him-4 result in pleiotropic defects during development and demonstrate tissue fragility. While previous studies in *C. elegans *have confirmed the localization of either transgenic HIM-4::GFP or endogenous C-terminally tagged HIM-4::mNeonGreen, only green or green/yellow fluorescent protein-tagged HIM-4 are available to the C. elegans community. Here, I used CRISPR/Cas9 technology to insert the far-red fluorescence protein mKate2 at the *him-4* genomic locus, and established worms expressing mKate2::HIM-4. As expected, localization of mkate2::HIM-4 at the rachis was observed at the L4 stage. In contrast to the localization of type IV collagen or nidogen such as major components of the basement membrane, mkate2::HIM-4 was polarized at the anterior part of the pharyngeal basement membrane. A unique polarized localization pattern of pharyngeal basement membrane is maintained throughout the L1–L4 stages.

**Figure 1. Fluorescence images of N-terminally endogenous mKate2-tagged HIM-4:**
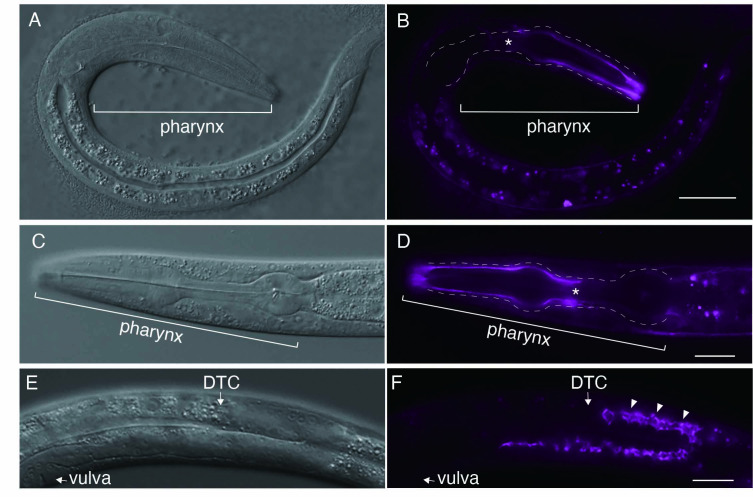
(A and B) Differential interference contrast (DIC) images of *him-4(xyz18[mkate2::HIM-4])* (A) and anterior localization of mkate2::HIM-4 to the pharyngeal basement membrane (B) at the L1 stage. Asterisks indicate the edge of anterior localization of mkate2::HIM-4. The white dotted lines outline the pharynx. (C and D) DIC images of *him-4(xyz18[mkate2::HIM-4])* (C) and anterior localization of mkate2::HIM-4 (D) at the L4 stage. Asterisks indicate the edge of anterior localization of mkate2::HIM-4. The white dotted lines outline the pharynx. **(E and F)** DIC images (E) and localization of mkate2::HIM-4 of the developing germline in *him-4(xyz18[mkate2::HIM-4])* (F) at the L4 stage*.* In F, white arrowheads mark the rachis that supports the development of the germline syncytium. Scale bars: 20 μm.

## Description

The gene *him-4* in *Caenorhabditis elegans* encodes hemicentin, which is evolutionally conserved (Vogel & Hedgecock, 2001). Hemicentins are characterized by multiple domains, including a conserved von Willebrand A domain, a long chain of immunoglobulin modules, a series of EGF-like modules, and a carboxyl-terminal fibulin-type module(Whittaker & Hynes, 2002).Despite being an extracellular matrix protein, mutant alleles of hemicentin exhibit both pleiotropic defects during development and tissue fragility (Hodgkin *et al.*, 1979; Vogel & Hedgecock, 2001). *HMCN1*, a human ortholog of *him-4,* is involved in age-related macular degeneration 1(Thompson *et al.*, 2007). Previous studies in *C. elegans* have shown that HIM-4 is secreted from the skeletal muscle and gonads and is recruited to specific extracellular sites using a transgenic strain (Vogel & Hedgecock, 2001). A recent study reported the construction of a C-terminally endogenous mNeonGreen-tagged *him-4* using CRISPR/Cas9 genome editing (Keeley *et al.*, 2020). Using the bright far-red fluorescence protein mKate2, I generated a homozygous mkate2::HIM-4strain, where mKate2 is inserted after the 109th glycine residue of the *him-4* genomic locus via CRISPR/Cas9 genome editing. As expected, localization of mkate2::HIM-4 to the pharyngeal basement membrane was observed at the L1 stage (Figure 1B). In contrast to the uniform localization on the basement membrane of type IV collagen or nidogen, which are major components of the basement membrane, that of mkate2::HIM-4 was polarized at the anterior part of the pharyngeal basement membrane (Kang & Kramer, 2013; Matsuo *et al.*, 2019)(Figure 1B). A unique polarized localization pattern was also observed in the pharyngeal basement membrane at stage L4 (Figure 1D). These observations indicate that polarized HIM-4 localization in the anterior part of the pharyngeal BM is maintained throughout the L1–L4 stages, although it remains unclear whether polarized localization is associated with pharynx function. The localization was also observed at the rachis during gonadal development (Figure 1F) at the L4 stage; this observation was consistent with that of previous reports (Vogel & Hedgecock, 2001). As the repellent behavior of *C. elegans* is observed after exposure to blue light (488 nm) that excites GFP but not after exposure to light (588 nm) that excites red fluorescent protein(Ward *et al.*, 2008), live imaging may be useful for visualizing mkate2::HIM-4 localization *in vivo*.

## Methods

Animals were cultured on standard NGM plates with *E. coli* (OP50) at 20 °C. The N2 (Bristol) strain was used as the injection strain. To generate a genome-edited mKate2 knock-in *him-4* locus, the pDD287 vector was used as the repair template. pDD287 was modified to generate the N-terminal mkate2::self-exiting cassette system repair template using the following primers: 5ʹ *him-4* fwd AACGACGGCCAGTCGGATGACAAAAATGACCCTACT, 5ʹ arm *him-4* rev GGCTCCCGATGCTCCTCCGTGCACGTACACTTTACTG, 3ʹ arm *him-4* fwd AGCGAGGAAGACTTGGGAGGTGATTGTCCAGAGAAG, and 3ʹ arm *him-4* rev CTATGACCATGTTATCATAAATGAATGAGGACGGTAA. Underlines highlight homology arms of *him-4* gene. The DNA sequences of all the constructed plasmids were confirmed via Sanger sequencing. The pDD162 (Peft-3::Cas9 + Empty sgRNA) vector (Addgene plasmid # 47549) was used for Cas9 expression (Dickinson *et al.*, 2013). The sgRNA plasmid was derived from Addgene Plasmid 46169. For direct cleavage of the target sequence, the following sgRNA sequence was used for *him-4*: 5ʹ AAAGTGTACGTGCACGGA/GG 3ʹ. To prevent re-digestion after knock-in of the *him-4* locus, a fluorescence mKate2 tag was inserted immediately before the position of the PAM sequence for the sgRNA sequence (/) at *him-4*. The sgRNA vectors were microinjected together with 50 ng/µL pDD162, 50 ng/µL sur-5::gfp, and 50 ng/µL repair template in N2 animals. Single *mkate2::HIM-4* was isolated based on the roller phenotype and sur-5::GFP expression, and the SEC was excised as described previously (Dickinson & Goldstein, 2016). All images were acquired using an Axiocam 506 mono, mounted on a Zeiss AxioImage M2 microscope, equipped with 20× or 40× Plan Apochromat objective lenses, and controlled using ZEN 2.3 pro (Zeiss). All images were optimized and superimposed using Photoshop 2021 (Adobe Systems).

Strain: IHR-187 *xyz18[mkate2::HIM-4]*X

It will be made available at the CGC.
